# Supplementary material for: Dynamic balance of myoplasmic energetics, redox state and protons in a fast‐twitch oxidative glycolytic skeletal muscle fibre
Source: J Physiol. 2026 Feb 14;604(5):1840–71. doi: 10.1113/JP289702 (PMC12953025; doi:10.1113/JP289702)
Supplement: Supplementary file 2 — Data S1 [file TJP-604-1840-s002.pdf]

Supplementary Material for "Dynamic balance of myoplasmic energetics, redox state and protons in a fast-twitch oxidative glycolytic skeletal muscle fiber"

Disch Jana[1], Jeneson Jeroen A.L.[2,3], Beard Daniel A.[4], Röhrle Oliver[1,5] and Thomas Klotz[1]

[1]Institute for Modelling and Simulation of Biomechanical Systems, University of Stuttgart, Germany
[2]Center for Child Development and Exercise, Wilhelmina Children's Hospital, University Medical Center Utrecht, the Netherlands
[3]Biomedical MR Research Lab, Amsterdam University Medical Center—site AMC, the Netherlands
[4]Department of Molecular and Integrative Physiology, University of Michigan, USA
[5]Stuttgart Centre for Simulation Science (SC SimTech), University of Stuttgart, Germany

November 2025

S1 Mathematical model

A detailed schematic overview of the computational model is shown in Fig. S1. Each flux is modelled as a function of metabolite concentrations associated with the corresponding biochemical reaction. If not disclosed otherwise, biochemical reactions are reversible and thermodynamically constrained by the Haldane relation as described in Lambeth and Kushmerick (2002). Exceptions are the kinetic equations of ATPase, PFK, and OxPhos. We used the Biochemical Simulation Environment, BISEN (Medical College of Wisconsin, WI, USA) (Vanlier et al., 2009), to generate the system of differential equations. The effects of proton binding on the thermodynamics and kinetics of biochemical reactions are accounted for as proposed by Vinnakota et al. (2009). The hereto necessary reference reactions to each biochemical reaction are listed in Table S1. The forward direction was defined as the reaction proceeding from left to right. The V^k_{max} values are modelled as functions of pH using an empirical approach, for enzyme activities with known pH dependence over the physiological range. Thereby, the maximal enzyme activity at a specific pH (V^k_{app}) is the product of the maximal activities (V^k_{max}) and a normalized algebraic function with its maximum at pH optimum (Vinnakota et al., 2006, 2010).

S1.1 Flux expressions

S1.1.1 ATP hydrolysis

We modelled ATPase activity as an irreversible reaction, entering the model as a boundary flux. The reaction flux is the superposition of a fixed basal rate $V^{\mathrm{ATPase}}_{\mathrm{basal}}$ (Blei et al., 1993) and a step function with variable amplitude $V^{\mathrm{ATPase}}_{\mathrm{act.}}$ depending on the work rate (WR_j) in a time interval $A_j = \{\, t \in \mathbb{R} \mid t_j \leq t < t_{j+1}\}$:

$$J^{\mathrm{ATPase}} = V^{\mathrm{ATPase}}_{\mathrm{basal}} + \sum_{j=0}^{N_A} V^{\mathrm{ATPase}}_{\mathrm{act.}}(\mathrm{WR}_j) \cdot f_{A_j}(t) \ , \tag{S1a}$$

$$f_{A_j}(t) = \begin{cases} 0 & \text{if } t \notin A_j \ , \\ 1 & \text{if } t \in A_j \ . \end{cases} \tag{S1b}$$

Therein, $N_A \geq 0$ denotes the number of simulated exercise phases and f_{A_j} denotes the interval indicator function. $V^{\mathrm{ATPase}}_{\mathrm{act.}} \in \mathbb{R}$ is the model input variable.

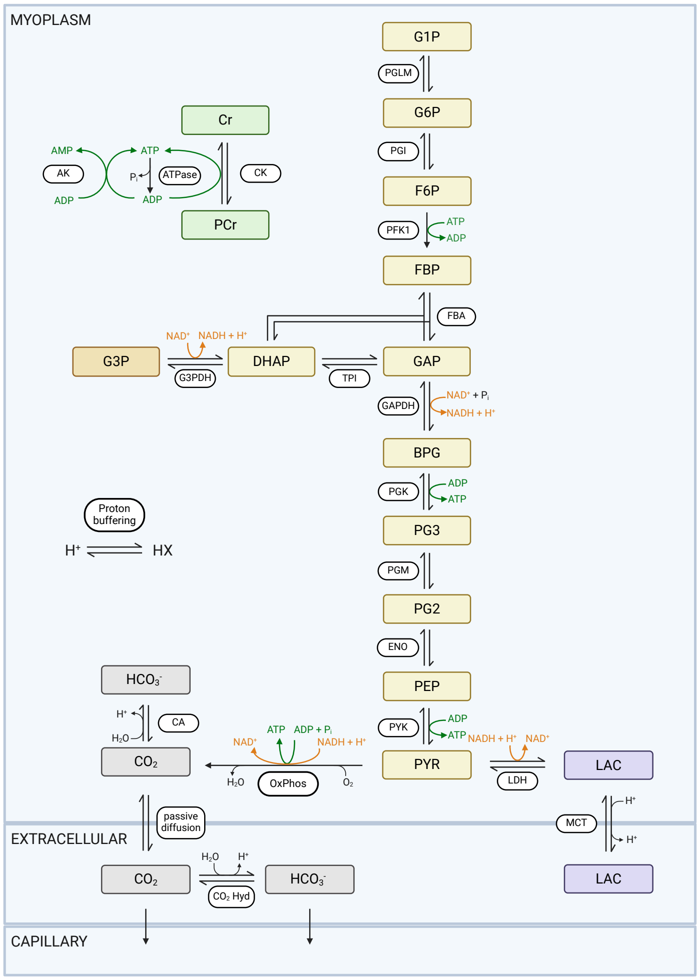

Fig S1. Schematic overview of the computational model
The model consists of a myoplasmic compartment (myoplasm), a compartment representing the interstitial fluid (extracellular), and a capillary compartment (capillary). The metabolic reaction network comprises a lumped ATP hydrolysis rate (ATPase) balanced by ATP re-synthesis through (i.) the buffer reactions catalysed by creatine kinase (CK) and adenylate kinase (AK), (ii.) glyco(geno)lysis with every enzyme-catalysed reaction modelled individually starting from phosphogluco mutase (PGLM) to lactate dehydrogenase (LDH), and (iii.) a phenomenological description of oxidative phosphorylation (OxPhos). Additional redox buffering through the glycerol-3-phosphate dehydrogenase (G3PDH) reaction and proton buffering through the carbonic anhydrase (CA) reaction and an intrinsic cellular proton buffer are considered. The model also accounts for facilitated lactate transport via monocarboxylate transport (MCT4) and CO_2 diffusion between the myoplasmic and extracellular compartments, as well as CO_2 and bicarbonate washout from the extracellular space into the capillary compartment. The figure was created using BioRender.com.

Table S1. Reference reactions.

Enzyme (EC No.)	Enzyme abbreviation	Reference reaction	n^k
**Myoplasmic compartment**			
ATP hydrolysis	ATPase	$ATP^{4-} + H_2O \rightarrow ADP^{3-} + HPi^{2-} + H^+$	1
Creatine kinase (2.7.3.2)	CK	$HPCr^{2-} + ADP^{3-} + H^+ \rightleftarrows HCr^0 + ATP^{4-}$	-1
Adenylate kinase (2.7.4.3)	AK	$ATP^{4-} + AMP^{2-} \rightleftarrows 2ADP^{3-}$	0
Phosphogluco mutase (2.7.1.41)	PGLM	$G1P^{2-} \rightleftarrows G6P^{2-}$	0
Phosphogluco isomerase (5.3.1.9)	PGI	$G6P^{2-} \rightleftarrows F6P^{2-}$	0
Phosphofructokinase (2.7.1.11)	PFK	$F6P^{2-} + ATP^{4-} \rightarrow FBP^{4-} + ADP^{3-} + H^+$	1
Fructose-bisphosphate aldolase (4.1.2.13)	FBA	$FBP^{4-} \rightleftarrows DHAP^{2-} + GAP^{2-}$	0
Triosephosphate isomerase (5.3.1.1)	TPI	$GAP^{2-} \rightleftarrows DHAP^{2-}$	0
Glyceraldehyde-3-phosphate dehydrogenase (1.2.1.12)	GAPDH	$GAP^{2-} + HPi^{2-} + NAD^- \rightleftarrows BPG^{4-} + NADH^{2-} + H^+$	1
Phosphoglycerate kinase (2.7.2.3)	PGK	$BPG^{4-} + ADP^{2-} \rightleftarrows PG3^{3-} + ATP^{4-}$	0
Phosphoglycerate mutase (5.4.2.1)	PGYM	$PG3^{3-} \rightleftarrows PG2^{3-}$	0
Enolase (4.2.1.11)	ENO	$PG2^{3-} \rightleftarrows PEP^{3-} + H_2O$	0
Pyruvate kinase (2.7.1.40)	PYK	$PEP^{3-} + ADP^{3-} + H^+ \rightleftarrows PYR^- + ATP^{4-}$	-1
Lactate dehydrogenase (1.1.1.27)	LDH	$PYR^- + NADH^{2-} + H^+ \rightleftarrows LAC^- + NAD^-$	-1
Glycerol-3-phosphate dehydrogenase (1.1.1.8)	G3PDH	$G3P^{2-} + NAD^- \rightleftarrows DHAP^{2-} + NADH^{2-} + H^+$	1
Oxidative phosphorylation	OxPhos	see Eqn. (S16)	-18
Carbonic anhydrase	CA	$CO_2 + H_2O \rightleftarrows HCO_3^- + H^+$	1
**Extracellular compartment**			
CO_2 hydrolysis	CO_2 Hyd	$CO_2 + H_2O \rightleftarrows HCO_3^- + H^+$	1

S1.1.2 ATP buffer reactions

Creatine kinase, CK

$$J^{\rm CK} = \frac{\left(V^{\rm CK}_{\rm max,f}\cdot\frac{[\rm ADP]\cdot[\rm PCr]}{K_{\rm iADP}\cdot K_{\rm PCr}}\right)-\left(V^{\rm CK}_{\rm max,r}\cdot\frac{[\rm ATP]\cdot[\rm Cr]}{K_{\rm iATP}\cdot K_{\rm Cr}}\right)}{1+\frac{[\rm ADP]}{K_{\rm iADP}}+\frac{[\rm PCr]}{K_{\rm iPCr}}+\frac{[\rm ADP]\cdot[\rm PCr]}{K_{\rm iADP}\cdot K_{\rm PCr}}+\frac{[\rm ATP]}{K_{\rm iATP}}+\frac{[\rm ATP]\cdot[\rm Cr]}{K_{\rm iATP}\cdot K_{\rm Cr}}}\ , \tag{S2a}$$

$$V^{\rm CK}_{\rm max,f} = \frac{V^{\rm CK}_{\rm max,r}\cdot K_{\rm iADP}\cdot K_{\rm PCr}\cdot K^{\rm CK}_{\rm app}}{K_{\rm iATP}\cdot K_{\rm Cr}}\ . \tag{S2b}$$

Adenylate kinase, AK

$$J^{\rm AK} = \frac{\left(V^{\rm AK}_{\rm max,f}\cdot\frac{[\rm ATP]\cdot[\rm AMP]}{K_{\rm ATP}\cdot K_{\rm AMP}}\right)-\left(V^{\rm AK}_{\rm max,r}\cdot\frac{[\rm ADP]^2}{K_{\rm ADP}{}^2}\right)}{1+\frac{[\rm ATP]}{K_{\rm ATP}}+\frac{[\rm AMP]}{K_{\rm AMP}}+\frac{[\rm ATP]\cdot[\rm AMP]}{K_{\rm ATP}\cdot K_{\rm AMP}}+\frac{2\cdot[\rm ADP]}{K_{\rm ADP}}+\frac{[\rm ADP]^2}{K_{\rm ADP}{}^2}}\ , \tag{S3a}$$

$$V^{\rm AK}_{\rm max,r} = \frac{V^{\rm AK}_{\rm max,f}\cdot K^2_{\rm ADP}}{K_{\rm ATP}\cdot K_{\rm AMP}\cdot K^{\rm AK}_{\rm app}}\ . \tag{S3b}$$

S1.1.3 Glycolytic pathway

Phosphoglucomutase, PGLM

$$J^{\mathrm{PGLM}} = \frac{\left(V_{\mathrm{app,f}}^{\mathrm{PGLM}} \cdot \frac{[\mathrm{G1P}]}{K_{\mathrm{G1P}}}\right) - \left(V_{\mathrm{max,r}}^{\mathrm{PGLM}} \cdot \frac{[\mathrm{G6P}]}{K_{\mathrm{G6P}}}\right)}{1 + \frac{[\mathrm{G1P}]}{K_{\mathrm{G1P}}} + \frac{[\mathrm{G6P}]}{K_{\mathrm{G6P}}}} \;, \tag{S4a}$$

$$V_{\mathrm{max,r}}^{\mathrm{PGLM}} = \frac{V_{\mathrm{app,f}}^{\mathrm{PGLM}} \cdot K_{\mathrm{G6P}}}{K_{\mathrm{G1P}} \cdot K_{\mathrm{app}}^{\mathrm{PGLM}}} \;, \tag{S4b}$$

$$V_{\mathrm{app,f}}^{\mathrm{PGLM}} = V_{\mathrm{max,f}}^{\mathrm{PGLM}} \cdot \left(\frac{1.329}{1 + 10^{6.64-\mathrm{pH}} + 10^{\mathbf{pH}-8.36}}\right) \;. \tag{S4c}$$

Phosphoglucoisomerase, PGI

$$J^{\mathrm{PGI}} = \frac{\left(V_{\mathrm{max,f}}^{\mathrm{PGI}} \cdot \frac{[\mathrm{G6P}]}{K_{\mathrm{G6P}}}\right) - \left(V_{\mathrm{app,r}}^{\mathrm{PGI}} \cdot \frac{[\mathrm{F6P}]}{K_{\mathrm{F6P}}}\right)}{1 + \frac{[\mathrm{G6P}]}{K_{\mathrm{G6P}}} + \frac{[\mathrm{F6P}]}{K_{\mathrm{F6P}}}} \;, \tag{S5a}$$

$$V_{\mathrm{max,f}}^{\mathrm{PGI}} = \frac{V_{\mathrm{app,r}}^{\mathrm{PGI}} \cdot K_{\mathrm{G6P}} \cdot K_{\mathrm{app}}^{\mathrm{PGI}}}{K_{\mathrm{F6P}}} \;, \tag{S5b}$$

$$V_{\mathrm{app,r}}^{\mathrm{PGI}} = V_{\mathrm{max,r}}^{\mathrm{PGI}} \cdot (0.4203 \cdot \mathrm{pH} - 2.3731) \;. \tag{S5c}$$

Phosphofructokinase, PFK The kinetic model description for PFK originates from Waser et al. (1983) and includes an adaptation to the $K_{\mathrm{i}}^{\mathrm{ATP}}$ term by Connett (1989) to account for competitive ADP and AMP activation to the ATP inhibition. The term was further adapted by dismissing a competitive activation via Pi, which was originally included in Connett's adaptation, and considering the deinhibition effect of ADP instead of MgADP (Schmitz et al., 2013). All parameters of the PFK model are provided in Table S2. Note that $V_{\mathrm{max}}^{\mathrm{PFK}}$ is not modelled as a function of pH.

$$
\begin{aligned}
J^{\mathrm{PFK}} &= V_{\max}^{\mathrm{PFK}} \cdot \frac{k \cdot \mathrm{NUM}}{E + E_{\mathrm{A}} + E_{\mathrm{AB}} + E_{\mathrm{BA}}} \\
\mathrm{NUM} &= Q_1 \cdot \left(1 + \frac{\bar{Q}}{c_1 c_2} + Q_6\right) + Q_2 \cdot \left(1 + \frac{\bar{Q}}{c_1 c_2} + Q_5\right) \cdot \left(1 + \frac{\bar{Q}}{c_1 c_2}\right) \\
E &= \left(1 + \frac{\bar{Q}}{c_1 c_2} + Q_5\right) \cdot \left(1 + \frac{\bar{Q}}{c_1 c_2} + Q_6\right) \cdot (1 + Q_7) \\
E_{\mathrm{A}} &= Q_1 \cdot \left(1 + \frac{\bar{Q}}{c_1 c_2} + Q_6\right) \cdot Q_3 \cdot \left(1 + \frac{\bar{Q}}{c_1}\right) \\
E_{\mathrm{B}} &= Q_2 \cdot \left(1 + \frac{\bar{Q}}{c_1 c_2} + Q_5\right) \cdot Q_4 \cdot \left(1 + \frac{\bar{Q}}{c_2}\right) \\
E_{\mathrm{BA}} + E_{\mathrm{AB}} &= \mathrm{NUM} \cdot \left(1 + \frac{\bar{Q}}{c_1 c_2}\right) \\
Q_1 &= \frac{[\mathrm{F6P}]}{K_{\mathrm{m}_1}^{\mathrm{F6P}}} \cdot \left(1 + \frac{Q_7}{c_1}\right) \\
Q_2 &= \frac{[\mathrm{MgATP}]}{K_{\mathrm{m}_1}^{\mathrm{MgATP}}} \cdot \left(1 + \frac{Q_7}{c_2}\right) \\
Q_3 &= \left(1 + \frac{\bar{Q}}{c_1}\right) \cdot \left(\frac{K_{\mathrm{m}_2}^{\mathrm{MgATP}}}{[\mathrm{MgATP}]}\right) \\
Q_4 &= \left(1 + \frac{\bar{Q}}{c_2}\right) \cdot \left(\frac{K_{\mathrm{m}_2}^{\mathrm{F6P}}}{[\mathrm{F6P}]}\right) \\
Q_5 &= \left(\frac{K_{\mathrm{i}}^{\mathrm{F6P}}}{[\mathrm{F6P}]}\right) \cdot \frac{[\mathrm{F6P}]}{K_{\mathrm{m}_1}^{\mathrm{F6P}}} \cdot (1 + \bar{Q}) \cdot Q_3 \\
Q_6 &= \left(\frac{Q_{\mathrm{i}}^{\mathrm{MgATP}}}{[\mathrm{MgATP}]}\right) \cdot \frac{[\mathrm{MgATP}]}{K_{\mathrm{m}_1}^{\mathrm{MgATP}}} \cdot (1 + \bar{Q}) \cdot Q_4 \\
Q_7 &= \frac{[\mathrm{H}^+]}{K_a} \cdot \left[1 + \left(\frac{[\mathrm{ATPH}]}{Q_{\mathrm{i}}^{\mathrm{ATPH}}}\right)^4\right] \\
\bar{Q} &= Q_7 \cdot \frac{\left(1 + \frac{1}{c_1}\frac{[\mathrm{F6P}]}{K_{\mathrm{m}_1}^{\mathrm{F6P}}}\right)^3 \cdot \left(1 + \frac{1}{c_2}\frac{[\mathrm{MgATP}]}{K_{\mathrm{m}_1}^{\mathrm{MgATP}}}\right)^3}{\left(1 + \frac{[\mathrm{F6P}]}{K_{\mathrm{m}_1}^{\mathrm{F6P}}}\right)^3 \cdot \left(1 + \frac{[\mathrm{MgATP}]}{K_{\mathrm{m}_1}^{\mathrm{MgATP}}}\right)^3} \\
Q_{\mathrm{i}}^{\mathrm{MgATP}} &= K_{\mathrm{i}}^{\mathrm{MgATP}} \cdot \left(1 + \frac{[\mathrm{ADP}]}{K_{\mathrm{ADP}}^{\mathrm{PFK}}} + \frac{[\mathrm{AMP}]}{K_{\mathrm{AMP}}^{\mathrm{PFK}}}\right) \\
Q_{\mathrm{i}}^{\mathrm{ATPH}} &= K_{\mathrm{i}}^{\mathrm{ATPH}} \cdot \left(1 + \frac{[\mathrm{ADP}]}{K_{\mathrm{ADP}}^{\mathrm{PFK}}} + \frac{[\mathrm{AMP}]}{K_{\mathrm{AMP}}^{\mathrm{PFK}}}\right)
\end{aligned} \tag{S6}
$$

Fructose-bisphosphate aldolase, FBA

$$
J^{\mathrm{FBA}} = \frac{\left(V_{\mathrm{app,f}}^{\mathrm{FBA}} \cdot \frac{[\mathrm{FBP}]}{K_{\mathrm{FBP}}}\right) - \left(V_{\mathrm{max,r}}^{\mathrm{FBA}} \cdot \frac{[\mathrm{DHAP}] \cdot [\mathrm{GAP}]}{K_{\mathrm{DHAP}} \cdot K_{\mathrm{GAP}}}\right)}{1 + \frac{[\mathrm{FBP}]}{K_{\mathrm{FBP}}} + \frac{[\mathrm{DHAP}]}{K_{\mathrm{DHAP}}} + \frac{[\mathrm{GAP}]}{K_{\mathrm{GAP}}}}\ , \tag{S7a}
$$

$$
V_{\mathrm{max,r}}^{\mathrm{FBA}} = \frac{V_{\mathrm{app,f}}^{\mathrm{FBA}} \cdot K_{\mathrm{DHAP}} \cdot K_{\mathrm{GAP}}}{K_{\mathrm{FBP}} \cdot K_{\mathrm{app}}^{\mathrm{FBA}}}\ , \tag{S7b}
$$

$$
V_{\mathrm{app,f}}^{\mathrm{FBA}} = V_{\mathrm{max,f}}^{\mathrm{FBA}} \cdot \left(0.5 + 0.5 \cdot \sin\left(\frac{\pi}{3.2} \cdot (\mathrm{pH} + 0.25)\right)\right)\ . \tag{S7c}
$$

Triosephosphate isomerase, TPI

$$J^{\mathrm{TPI}} = \frac{\left(V_{\mathrm{max,f}}^{\mathrm{TPI}} \cdot \frac{[\mathrm{GAP}]}{K_{\mathrm{GAP}}}\right) - \left(V_{\mathrm{max,r}}^{\mathrm{TPI}} \cdot \frac{[\mathrm{DHAP}]}{K_{\mathrm{DHAP}}}\right)}{1 + \frac{[\mathrm{GAP}]}{K_{\mathrm{GAP}}} + \frac{[\mathrm{DHAP}]}{K_{\mathrm{DHAP}}}} , \tag{S8a}$$

$$V_{\mathrm{max,r}}^{\mathrm{TPI}} = \frac{V_{\mathrm{max,f}}^{\mathrm{TPI}} \cdot K_{\mathrm{DHAP}}}{K_{\mathrm{GAP}} \cdot K_{\mathrm{app}}^{\mathrm{TPI}}} . \tag{S8b}$$

Glyceraldehyde-3-phosphate dehydrogenase, GAPDH

$$J^{\mathrm{GAPDH}} = \frac{\left(V_{\mathrm{app,f}}^{\mathrm{GAPDH}} \cdot \frac{[\mathrm{GAP}]\cdot[\mathrm{NAD}]\cdot[\mathrm{Pi}]}{K_{\mathrm{GAP}}\cdot K_{\mathrm{NAD}}\cdot K_{\mathrm{Pi}}}\right) - \left(V_{\mathrm{max,r}}^{\mathrm{GAPDH}} \cdot \frac{[\mathrm{BPG}]\cdot[\mathrm{NADH}]}{K_{\mathrm{BPG}}\cdot K_{\mathrm{NADH}}}\right)}{D^{\mathrm{GAPDH}}} , \tag{S9a}$$

$$\begin{aligned} D^{\mathrm{GAPDH}} = {} & 1 + \frac{[\mathrm{GAP}]}{K_{\mathrm{GAP}}} + \frac{[\mathrm{NADH}]}{K_{\mathrm{NADH}}} + \frac{[\mathrm{Pi}]}{K_{\mathrm{Pi}}} + \frac{[\mathrm{GAP}] \cdot [\mathrm{NAD}]}{K_{\mathrm{GAP}} \cdot K_{\mathrm{NADH}}} + \\ & \frac{[\mathrm{GAP}] \cdot [\mathrm{NAD}] \cdot [\mathrm{Pi}]}{K_{\mathrm{GAP}} \cdot K_{\mathrm{NADH}} \cdot K_{\mathrm{Pi}}} + \frac{[\mathrm{BPG}]}{K_{\mathrm{BPG}}} + \frac{[\mathrm{NADH}]}{K_{\mathrm{NADH}}} + \frac{[\mathrm{BPG}] \cdot [\mathrm{NADH}]}{K_{\mathrm{BPG}} \cdot K_{\mathrm{NADH}}} , \end{aligned} \tag{S9b}$$

$$V_{\mathrm{max,r}}^{\mathrm{GAPDH}} = \frac{V_{\mathrm{app,f}}^{\mathrm{GAPDH}} \cdot K_{\mathrm{BPG}} \cdot K_{\mathrm{NADH}}}{K_{\mathrm{GAP}} \cdot K_{\mathrm{NAD}} \cdot K_{\mathrm{Pi}} \cdot K_{\mathrm{app}}^{\mathrm{GAPDH}}} , \tag{S9c}$$

$$V_{\mathrm{app,f}}^{\mathrm{GAPDH}} = V_{\mathrm{max,f}}^{\mathrm{GAPDH}} \cdot (0.007 \cdot \exp(0.8979 \cdot \mathrm{pH})) . \tag{S9d}$$

Phosphoglycerate kinase, PGK

$$J^{\mathrm{PGK}} = \frac{\left(V_{\mathrm{max,f}}^{\mathrm{PGK}} \cdot \frac{[\mathrm{BPG}]\cdot[\mathrm{ADP}]}{K_{\mathrm{BPG}}\cdot K_{\mathrm{ADP}}}\right) - \left(V_{\mathrm{max,r}}^{\mathrm{PGK}} \cdot \frac{[\mathrm{PG3}]\cdot[\mathrm{ATP}]}{K_{\mathrm{PG3}}\cdot K_{\mathrm{ATP}}}\right)}{1 + \frac{[\mathrm{BPG}]}{K_{\mathrm{BPG}}} + \frac{[\mathrm{ADP}]}{K_{\mathrm{ADP}}} + \frac{[\mathrm{BPG}]\cdot[\mathrm{ADP}]}{K_{\mathrm{BPG}}\cdot K_{\mathrm{ADP}}} + \frac{[\mathrm{PG3}]}{K_{\mathrm{PG3}}} + \frac{[\mathrm{ATP}]}{K_{\mathrm{ATP}}} + \frac{[\mathrm{PG3}]\cdot[\mathrm{ATP}]}{K_{\mathrm{PG3}}\cdot K_{\mathrm{ATP}}}} , \tag{S10a}$$

$$V_{\mathrm{max,f}}^{\mathrm{PGK}} = \frac{V_{\mathrm{max,r}}^{\mathrm{PGK}} \cdot K_{\mathrm{BPG}} \cdot K_{\mathrm{ADP}} \cdot K_{\mathrm{app}}^{\mathrm{PGK}}}{K_{\mathrm{PG3}} \cdot K_{\mathrm{ATP}}} . \tag{S10b}$$

Phosphoglycerate mutase, PGYM

$$J^{\mathrm{PGYM}} = \frac{\left(V_{\mathrm{app,f}}^{\mathrm{PGYM}} \cdot \frac{[\mathrm{PG3}]}{K_{\mathrm{PG3}}}\right) - \left(V_{\mathrm{max,r}}^{\mathrm{PGYM}} \cdot \frac{[\mathrm{PG2}]}{K_{\mathrm{PG2}}}\right)}{1 + \frac{[\mathrm{PG3}]}{K_{\mathrm{PG3}}} + \frac{[\mathrm{PG2}]}{K_{\mathrm{PG2}}}} , \tag{S11a}$$

$$V_{\mathrm{max,r}}^{\mathrm{PGYM}} = \frac{V_{\mathrm{app,f}}^{\mathrm{PGYM}} \cdot K_{\mathrm{PG2}}}{K_{\mathrm{PG3}} \cdot K_{\mathrm{app}}^{\mathrm{PGYM}}} , \tag{S11b}$$

$$V_{\mathrm{app,f}}^{\mathrm{PGYM}} = V_{\mathrm{max,f}}^{\mathrm{PGYM}} \cdot (0.4444 \cdot \mathrm{pH} - 2.2407) . \tag{S11c}$$

Enolase, ENO

$$J^{\mathrm{ENO}} = \frac{\left(V_{\mathrm{max,f}}^{\mathrm{ENO}} \cdot \frac{[\mathrm{PG2}]}{K_{\mathrm{PG2}}}\right) - \left(V_{\mathrm{max,r}}^{\mathrm{ENO}} \cdot \frac{[\mathrm{PEP}]}{K_{\mathrm{PEP}}}\right)}{1 + \frac{[\mathrm{PG2}]}{K_{\mathrm{PG2}}} + \frac{[\mathrm{PEP}]}{K_{\mathrm{PEP}}}} , \tag{S12a}$$

$$V_{\mathrm{max,r}}^{\mathrm{ENO}} = \frac{V_{\mathrm{max,f}}^{\mathrm{ENO}} \cdot K_{\mathrm{PEP}}}{K_{\mathrm{PG2}} \cdot K_{\mathrm{app}}^{\mathrm{ENO}}} . \tag{S12b}$$

Pyruvate kinase, PYK

$$J^{\mathrm{PYK}} = \frac{\left(V_{\mathrm{app,f}}^{\mathrm{PYK}} \cdot \frac{[\mathrm{PEP}]\cdot[\mathrm{ADP}]}{K_{\mathrm{PEP}}\cdot K_{\mathrm{ADP}}}\right) - \left(V_{\mathrm{max,r}}^{\mathrm{PYK}} \cdot \frac{[\mathrm{PYR}]\cdot[\mathrm{ATP}]}{K_{\mathrm{PYR}}\cdot K_{\mathrm{ATP}}}\right)}{1 + \frac{[\mathrm{PEP}]}{K_{\mathrm{PEP}}} + \frac{[\mathrm{ADP}]}{K_{\mathrm{ADP}}} + \frac{[\mathrm{PEP}]\cdot[\mathrm{ADP}]}{K_{\mathrm{PEP}}\cdot K_{\mathrm{ADP}}} + \frac{[\mathrm{PYR}]}{K_{\mathrm{PYR}}} + \frac{[\mathrm{ATP}]}{K_{\mathrm{ATP}}} + \frac{[\mathrm{PYR}]\cdot[\mathrm{ATP}]}{K_{\mathrm{PYR}}\cdot K_{\mathrm{ATP}}}} \ , \tag{S13a}$$

$$V_{\mathrm{max,r}}^{\mathrm{PYK}} = \frac{V_{\mathrm{app,f}}^{\mathrm{PYK}} \cdot K_{\mathrm{ATP}} \cdot K_{\mathrm{PYR}}}{K_{\mathrm{PEP}} \cdot K_{\mathrm{ADP}} \cdot K_{\mathrm{app}}^{\mathrm{PYK}}} \ , \tag{S13b}$$

$$V_{\mathrm{app,f}}^{\mathrm{PYK}} = \left(\frac{V_{\mathrm{max,f}}^{\mathrm{PYK}}}{1 + 10^{\mathrm{pH}-8.2}}\right) \ . \tag{S13c}$$

Lactate dehydrogenase, LDH

$$J^{\mathrm{LDH}} = \frac{\left(V_{\mathrm{app,f}}^{\mathrm{LDH}} \cdot \frac{[\mathrm{PYR}]\cdot[\mathrm{NADH}]}{K_{\mathrm{PYR}}\cdot K_{\mathrm{NADH}}}\right) - \left(V_{\mathrm{max,r}}^{\mathrm{LDH}} \cdot \frac{[\mathrm{LAC}]\cdot[\mathrm{NAD}]}{K_{\mathrm{LAC}}\cdot K_{\mathrm{NAD}}}\right)}{1 + \frac{[\mathrm{PYR}]}{K_{\mathrm{PYR}}} + \frac{[\mathrm{NADH}]}{K_{\mathrm{NADH}}} + \frac{[\mathrm{PYR}]\cdot[\mathrm{NADH}]}{K_{\mathrm{PYR}}\cdot K_{\mathrm{NADH}}} + \frac{[\mathrm{LAC}]}{K_{\mathrm{LAC}}} + \frac{[\mathrm{NAD}]}{K_{\mathrm{NAD}}} + \frac{[\mathrm{LAC}]\cdot[\mathrm{NAD}]}{K_{\mathrm{LAC}}\cdot K_{\mathrm{NAD}}}} \ , \tag{S14a}$$

$$V_{\mathrm{max,r}}^{\mathrm{LDH}} = \frac{V_{\mathrm{app,f}}^{\mathrm{LDH}} \cdot K_{\mathrm{LAC}} \cdot K_{\mathrm{NAD}}}{K_{\mathrm{PYR}} \cdot K_{\mathrm{NADH}} \cdot K_{\mathrm{app}}^{\mathrm{LDH}}} \ , \tag{S14b}$$

$$V_{\mathrm{app,f}}^{\mathrm{LDH}} = V_{\mathrm{max,f}}^{\mathrm{LDH}} \cdot (-0.1376 \cdot \mathrm{pH} + 1.745) \ . \tag{S14c}$$

S1.1.4 Redox buffer

Glycerol-3-phosphate dehydrogenase, G3PDH

$$J^{\mathrm{G3PDH}} = \frac{\left(V_{\mathrm{max,f}}^{\mathrm{G3PDH}} \cdot \frac{[\mathrm{G3P}]\cdot[\mathrm{NAD}]}{K_{\mathrm{G3P}}\cdot K_{\mathrm{NAD}}}\right) - \left(V_{\mathrm{max,r}}^{\mathrm{G3PDH}} \cdot \frac{[\mathrm{DHAP}]\cdot[\mathrm{NADH}]}{K_{\mathrm{DHAP}}\cdot K_{\mathrm{NADH}}}\right)}{\left(1 + \frac{[\mathrm{G3P}]}{K_{\mathrm{G3P}}} + \frac{[\mathrm{NADH}]}{K_{\mathrm{NADH}}}\right) \cdot \left(1 + \frac{[\mathrm{DHAP}]}{K_{\mathrm{DHAP}}} + \frac{[\mathrm{NAD}]}{K_{\mathrm{NAD}}}\right)} \ , \tag{S15a}$$

$$V_{\mathrm{max,f}}^{\mathrm{G3PDH}} = \frac{V_{\mathrm{max,r}}^{\mathrm{G3PDH}} \cdot K_{\mathrm{G3P}} \cdot K_{\mathrm{NAD}} \cdot K_{\mathrm{app}}^{\mathrm{G3PDH}}}{K_{\mathrm{DHAP}} \cdot K_{\mathrm{NADH}}} \ . \tag{S15b}$$

S1.1.5 The phenomenological OxPhos model

The reference reaction of OxPhos was defined as:

$$s_{\mathrm{PYR}} \cdot \mathrm{PYR}^- + s_{\mathrm{NADH}} \cdot \mathrm{NADH}^{2-} + s_{\mathrm{ADP}} \cdot \mathrm{ADP}^{3-} + s_{\mathrm{Pi}} \cdot \mathrm{HPO}_4^{2-} + s_{\mathrm{H}} \cdot \mathrm{H}^+ + s_{\mathrm{O_2}} \cdot \mathrm{O_2} \rightarrow s_{\mathrm{ATP}} \cdot \mathrm{ATP}^{4-} + s_{\mathrm{CO_2}} \cdot \mathrm{CO_2} + s_{\mathrm{H_2O}} \cdot \mathrm{H_2O} + s_{\mathrm{NAD}} \cdot \mathrm{NAD}^- \ . \tag{S16}$$

The stoichiometry is based on the following considerations. First, the respiratory quotient RQ (i.e., moles of CO_2 produced per consumed mole of O_2) was observed to be approximately 1.0 in resting and partially activated mouse fast twitch muscle (EDL; extensor digitorum longus) (Crow and Kushmerick, 1982). This reflects the primary breakdown of carbohydrates to generate acetyl-CoA. Thus, we only considered pyruvate as a substrate for the OxPhos model. The stoichiometry of the OxPhos reaction was calculated for the uptake of 1 pyruvate:

$$s_{\mathrm{PYR}} = 1 \ . \tag{S17}$$

Each pyruvate consumed is coupled to the generation of reducing agents in the form of 1 NADH from the pyruvate decarboxylation, 3 NADH from the citric acid cycle (TCA), and 1 QH_2 from the oxidation of succinate in complex II. In addition, myoplasmic NADH from glycogenolysis can be utilized in the Electron Transport Chain (ETC). Thereby, the stoichiometry factor of NADH in Eqn. (S16) was modelled as a function of myoplasmic NADH concentration to ensure mass conservation even under extreme conditions with limited (or no) availability of myoplasmic NADH:

$$s_{\mathrm{NADH}} = \frac{1}{1 + \left(\frac{K_{0.5}}{[\mathrm{NADH}]}\right)^{n_{\mathrm{H}}^{\mathrm{NADH}}}} \ . \tag{S18}$$

We assumed the half saturation concentration $K_{0.5}$ =0.1 µM and a Hill coefficient $n_{\mathrm{H}}^{\mathrm{NADH}} = 4$. Assuming the more efficient electron transport via malate-aspartate shuttle (Schantz et al., 1986), we end up with a total of $(4 + s_{\mathrm{NADH}})$ NADH and 1 QH_2 as reducing agents. Ten protons are pumped across the inner mitochondrial membrane for each oxidised NADH at complex I compared to six protons for every two electrons donated from QH_2 to complex II. This results in a total of $(46 \ + \ s_{\mathrm{NADH}} \cdot 10)$ protons being pumped per pyruvate molecule. The malate-aspartate shuttle is electrogenic (LaNoue and Schoolwerth, 1979), effectively consuming s_{NADH} protons per pyruvate, resulting in $(46 \ + \ s_{\mathrm{NADH}} \cdot 9)$ protons being pumped per pyruvate. The ATP synthase stoichiometry is 8/3 (Watt et al., 2010) plus one additional proton attributed to the transport of ATP (adenine nucleotide translocator, ANT) for a total cost of 11/3 protons per ATP. Considering the substrate-level production of 1 ATP derived from the guanosine triphosphate molecule (GTP) generated in the TCA cycle, we obtain:

$$s_{\mathrm{ATP}} = \frac{46 \ + \ s_{\mathrm{NADH}} \cdot 9}{11/3} + 1 \ . \tag{S19}$$

Note that we excluded the cost of 1 proton for the transport of the additional substrate-level ATP (ANT), as the simulations were not sensitive to small changes in proton stoichiometry (result not shown). By doing so, we obtain integer values for all stoichiometry factors under normal physiological conditions, i.e., with $s_{\mathrm{NADH}} = 1$.

The oxygen consumption flux is stoichiometrically equated to half of the rate of generation of electron donors:

$$s_{\mathrm{O_2}} = \frac{5 + s_{\mathrm{NADH}}}{2} \ . \tag{S20}$$

3 CO_2 are generated per pyruvate (1 from pyruvate decarboxylation and 2 from the TCA cycle):

$$s_{\mathrm{CO_2}} = 3 \ . \tag{S21}$$

The stoichiometry factors for NAD, ADP, and Pi are given by:

$$s_{\mathrm{NAD}} = s_{\mathrm{NADH}}, \tag{S22}$$

$$s_{\mathrm{ADP}} = s_{\mathrm{Pi}} = s_{\mathrm{ATP}} \ . \tag{S23}$$

Considering the uptake of 2 H_2O in the TCA cycle, the stoichiometry factor for H_2O is defined as:

$$s_{\mathrm{H_2O}} = (s_{\mathrm{ATP}} - 1) + 2 \cdot s_{\mathrm{O_2}} - 2 \ . \tag{S24}$$

Finally, all reference reactions in the model are balanced with respect to charge, leading to:

$$s_{\mathrm{H}} = 1 + s_{\mathrm{NADH}} + \frac{46 \ + \ s_{\mathrm{NADH}} \cdot 9}{11/3} + 1 \ . \tag{S25}$$

Under normal physiological conditions, s_{NADH} equals 1 and the stoichiometry of the OxPhos model is the one given in the main text (Eqn. 13, main text) with a maximal P/O_2 of 5.0 (amount of ATP produced per molecule of oxygen reduced by the ETC). With limited availability of myoplasmic NADH, the capacity for oxidative ATP production is reduced.
The kinetic equation can be found in the main text (Eqn. 14, main text).

S1.1.6 Carbon dioxide/bicarbonate buffering system

Carbonic Anhydrase

$$J^{\mathrm{CA}} = X^{\mathrm{CA}} \cdot \left(k_f \cdot [\mathrm{CO_2}] - k_b \cdot \left[\mathrm{H^+}\right] [\mathrm{HCO_3^-}]\right) \ . \tag{S26}$$

Uncatalyzed CO_2 hydration (extracellular compartment)

$$J^{\mathrm{CO2Hyd}} = \left(k_f \cdot [\mathrm{CO_2}] - k_b \cdot \left[\mathrm{H^+}\right] [\mathrm{HCO_3^-}]\right) \ . \tag{S27}$$

Table S2. Kinetic Parameters of the biochemical reaction models.

Model	Parameter	Parameter value [a]
**Myoplasmic compartment**		
**Creatine kinase**	$V_{\mathrm{max,r}}^{\mathrm{CK}}$	Table 1, main text
	K_{iADP}	1.35×10^{-4} M
	K_{PCr}	1.11×10^{-3} M
	K_{iATP}	3.5×10^{-3} M
	K_{Cr}	3.8×10^{-3} M
	K_{iPCr}	3.9×10^{-3} M
**Adenylate kinase**	$V_{\mathrm{max,f}}^{\mathrm{AK}}$	Table 1, main text
	K_{ATP}	2.7×10^{-4} M
	K_{AMP}	3.2×10^{-4} M
	K_{ADP}	3.5×10^{-4} M
**Phosphoglucomutase**	$V_{\mathrm{max,f}}^{\mathrm{PGLM}}$	Table 1, main text
	K_{G1P}	6.3×10^{-5} M
	K_{G6P}	3.0×10^{-5} M
**Phosphoglucoisomerase**	$V_{\mathrm{max,r}}^{\mathrm{PGI}}$	Table 1, main text
	K_{G6P}	4.8×10^{-4} M
	K_{F6P}	1.19×10^{-4} M
**Phosphofructokinase**	$V_{\mathrm{max,f}}^{\mathrm{PFK}}$	Table 1, main text
	$K_{\mathrm{m_1}}^{\mathrm{F6P}}$	68.49×10^{-6} M[b]
	$K_{\mathrm{m_2}}^{\mathrm{F6P}}$	58.70×10^{-6} M[b]
	$K_{\mathrm{m_1}}^{\mathrm{MgATP}}$	26.54×10^{-6} M[b]
	$K_{\mathrm{m_2}}^{\mathrm{MgATP}}$	37.81×10^{-6} M[b]
	$K_{\mathrm{i}}^{\mathrm{F6P}}$	4.33×10^{-6} M[b]
	$K_{\mathrm{i}}^{\mathrm{MgATP}}$	124.6×10^{-6} M[b]
	$K_{\mathrm{i}}^{\mathrm{ATPH}}$	0.649×10^{-6} M[b]
	K_{a}	0.0812×10^{-6} M[b]
	k	0.990[b]
	c_1	19.09[b]
	c_2	2.63[b]
	$K_{\mathrm{ADP}}^{\mathrm{PFK}}$	0.7×10^{-6} M[c]
	$K_{\mathrm{AMP}}^{\mathrm{PFK}}$	0.0123×10^{-6} M[c]
**Fructose-bisphosphate aldolase**	$V_{\mathrm{max,f}}^{\mathrm{FBA}}$	Table 1, main text
	K_{FBP}	5.0×10^{-5} M
	K_{DHAP}	2.0×10^{-3} M
	K_{GAP}	1.0×10^{-3} M
**Triosephophate isomerase**	$V_{\mathrm{max,f}}^{\mathrm{TPI}}$	Table 1, main text
	K_{GAP}	3.2×10^{-4} M
	K_{DHAP}	6.1×10^{-4} M
**Glyceraldehyde-3-phosphate dehydrogenase**	$V_{\mathrm{max,f}}^{\mathrm{GAPDH}}$	Table 1, main text
	K_{GAP}	2.5×10^{-6} M
	K_{NAD}	9.0×10^{-5} M
	K_{Pi}	2.9×10^{-4} M

Continuation of Table S2

	K_{BPG}	8.0×10^{-7} M
	K_{NADH}	3.3×10^{-6} M
**Phosphoglycerate kinase**	$V_{max,r}^{PGK}$	Table 1, main text
	K_{BPG}	2.0×10^{-3} M
	K_{ADP}	8.0×10^{-6} M
	K_{PG3}	1.2×10^{-3} M
	K_{ATP}	3.5×10^{-4} M
**Phosphoglycerate mutase**	$V_{max,f}^{PGYM}$	Table 1, main text
	K_{PG3}	2.0×10^{-4} M
	K_{PG2}	1.4×10^{-5} M
**Enolase**	$V_{max,f}^{ENO}$	Table 1, main text
	K_{PG2}	1.0×10^{-4} M
	K_{PEP}	3.7×10^{-4} M
**Pyruvate kinase**	$V_{max,f}^{PYK}$	Table 1, main text
	K_{PEP}	8.0×10^{-5} M
	K_{ADP}	3.0×10^{-4} M
	K_{PYR}	7.05×10^{-3} M
	K_{ATP}	1.13×10^{-3} M
**Lactate dehydrogenase**	$V_{max,f}^{LDH}$	Table 1, main text
	K_{PYR}	3.35×10^{-4} M
	K_{NADH}	2.0×10^{-6} M
	K_{LAC}	1.7×10^{-2} M
	K_{NAD}	8.49×10^{-4} M
**Glycerol-3-phosphate dehydrogenase**	$V_{max,r}^{G3PDH}$	Table 1, main text
	K_{G3P}	1.8×10^{-4} M
	K_{NAD}	1.2×10^{-5} M
	K_{DHAP}	2.2×10^{-4} M
	K_{NADH}	8.0×10^{-6} M
**OxPhos**	$V_{max,f}^{OxPhos}$	Table 2, main text
	K_{ADP}^{OxPhos}	Table 2, main text
	n_{H}^{OxPhos}	Table 2, main text
	K_{Pi}^{OxPhos}	1.0×10^{-3} M[d]
	K_{PYR}^{OxPhos}	0.15×10^{-3} M[e]
**Carbonic Anhydrase**	X^{CA}	950[f]
	k_f	0.0469 s^{-1}[f]
	k_b	9.89×10^4 $M^{-1}s^{-1}$[f]
**Extracellular compartment**		
**CO_2 Hydrolysis**	k_f	0.0469 s^{-1}[f]
	k_b	9.89×10^4 $M^{-1}s^{-1}$[f]

[a] If not declared otherwise, parameter values were obtained from Lambeth and Kushmerick (2002).
[b] values obtained from Waser et al. (1983).
[c] heuristically adjusted.
[d] value obtained from Chance and Connelly (1957).
[e] value obtained from Halestrap (1975).
[f] values obtained from Vinnakota et al. (2010).

S1.1.7 Transport mechanisms

The modelling framework includes facilitated lactate proton symport via MCT4 and passive CO_2 diffusion between the myoplasm compartment and extracellular space. The extracellular lactate concentration was clamped to 1 mM. CO_2 and bicarbonate are further removed from the extracellular space into a capillary

compartment. In the following, $[\mathrm{L}]_{\mathrm{my}}$, $[\mathrm{L}]_{\mathrm{ex}}$, and $[\mathrm{L}]_{\mathrm{cap}}$ indicate the concentrations of a metabolite L in the myoplasm, extracellular space, and the capillary compartment, respectively. $J^{k}_{\mathrm{my}\rightleftarrows\mathrm{ex}}$ indicates transport mechanism k between the myoplasmic and extracellular compartment and $J^{k}_{\mathrm{ex}\rightleftarrows\mathrm{cap}}$ indicates transport mechanism k between the extracellular space and the capillary compartment. The kinetic equations of all transport mechanisms were taken from a model of glycogenolytic and oxidative energy metabolism in resting skeletal muscle fibers (Vinnakota et al., 2010). The diffusion parameter of CO_2 ($\Phi^{\mathrm{CO_2}}_{\mathrm{diff}}$) and the washout parameter of CO_2 ($\Phi^{\mathrm{CO_2}}_{\mathrm{washout}}$) and bicarbonate transport ($\Phi^{\mathrm{HCO_3^-}}_{\mathrm{washout}}$) were adjusted in order to simulate actively contracting muscle fibers. The fixed CO_2 and bicarbonate concentrations in the capillary domain ($[\mathrm{CO_2}]_{\mathrm{cap}} = 1.5\,\mathrm{mM}$; $\left[\mathrm{HCO_3^-}\right]_{\mathrm{cap}} = 30\,\mathrm{mM}$) were taken from Vinnakota et al. (2010)[1]. Parameter values are listed in Table S3.

Monocarboxylate Transport, MCT4

$$J^{\mathrm{MCT}}_{\mathrm{my}\rightleftarrows\mathrm{ex}} = V^{\mathrm{MCT}}_{\max} \cdot \frac{\frac{[\mathrm{LAC}]_{\mathrm{my}}[\mathrm{H}]_{\mathrm{my}}}{K_{\mathrm{LAC}}K_{\mathrm{H}}} - \frac{[\mathrm{LAC}]_{\mathrm{ex}}[\mathrm{H}]_{\mathrm{ex}}}{K_{\mathrm{LAC}}K_{\mathrm{H}}}}{2 + \frac{[\mathrm{LAC}]_{\mathrm{my}}[\mathrm{H}]_{\mathrm{my}}}{K_{\mathrm{LAC}}K_{\mathrm{H}}} + \frac{[\mathrm{LAC}]_{\mathrm{my}}}{K_{\mathrm{LAC}}} + \frac{[\mathrm{H}]_{\mathrm{my}}}{K_{\mathrm{H}}} + \frac{[\mathrm{LAC}]_{\mathrm{ex}}[\mathrm{H}]_{\mathrm{ex}}}{K_{\mathrm{LAC}}K_{\mathrm{H}}} + \frac{[\mathrm{LAC}]_{\mathrm{ex}}}{K_{\mathrm{LAC}}} + \frac{[\mathrm{H}]_{\mathrm{ex}}}{K_{\mathrm{H}}}} \tag{S28}$$

$\mathbf{CO_2}$ diffusion

$$J^{\mathrm{CO_2}}_{\mathrm{my}\rightleftarrows\mathrm{ex}} = \Phi^{\mathrm{CO_2}}_{\mathrm{diff}} \cdot \Big([\mathrm{CO_2}]_{\mathrm{my}} - [\mathrm{CO_2}]_{\mathrm{ex}}\Big) \tag{S29}$$

$\mathbf{CO_2}$ washout

$$J^{\mathrm{CO_2}}_{\mathrm{ex}\rightleftarrows\mathrm{cap}} = -\Phi^{\mathrm{CO_2}}_{\mathrm{washout}} \cdot \left(1.5 \times 10^{-3} - [\mathrm{CO_2}]_{\mathrm{ex}}\right) \tag{S30}$$

$\mathbf{HCO_3^-}$ washout

$$J^{\mathrm{HCO_3^-}}_{\mathrm{ex}\rightleftarrows\mathrm{cap}} = -\Phi^{\mathrm{HCO_3^-}}_{\mathrm{washout}} \cdot \left(30 \times 10^{-3} - \left[\mathrm{HCO_3^-}\right]_{\mathrm{ex}}\right) \tag{S31}$$

S1.2 Differential equations

The model comprises the following set of differential equations. Not included in this list are the differential equations for protons and metal ions, which are obtained by the Biochemical Simulation Environment BISEN (Vanlier et al., 2009) as proposed by Vinnakota et al. (2009). The myoplasmic concentration of potassium, as well as the pH level in the extracellular and capillary compartments, are each clamped to the basal value reported in Table S4 for all simulations presented in this work.

Myoplasmic compartment

$$\frac{d\,[\mathrm{ATP}]}{dt} = \frac{-J^{\mathrm{PFK}} + J^{\mathrm{PGK}} + J^{\mathrm{PYK}} - J^{\mathrm{ATPase}} + J^{\mathrm{CK}} - J^{\mathrm{AK}} + 16 \cdot J^{\mathrm{OxPhos}}}{\nu^{\mathrm{w}}_{\mathrm{my}}} \tag{S32}$$

$$\frac{d\,[\mathrm{ADP}]}{dt} = \frac{J^{\mathrm{PFK}} - J^{\mathrm{PGK}} - J^{\mathrm{PYK}} + J^{\mathrm{ATPase}} - J^{\mathrm{CK}} + 2 \cdot J^{\mathrm{AK}} - 16 \cdot J^{\mathrm{OxPhos}}}{\nu^{\mathrm{w}}_{\mathrm{my}}} \tag{S33}$$

$$\frac{d\,[\mathrm{Pi}]}{dt} = \left(-J^{\mathrm{GAPDH}} + J^{\mathrm{ATPase}} - 16 \cdot J^{\mathrm{OxPhos}} - J^{\mathrm{PGLM}}\right) / \nu^{\mathrm{w}}_{\mathrm{my}} \tag{S34}$$

$$\frac{d\,[\mathrm{AMP}]}{dt} = \left(-J^{\mathrm{AK}}\right) / \nu^{\mathrm{w}}_{\mathrm{my}} \tag{S35}$$

[1]Available from: `https://models.physiomeproject.org/exposure/aa821a8dda7e55888bd486f8cbb68791/vinnakota_rusk_palmer_shankland_kushmeric_2010_edl.cellml/view`; Accessed: 2025-01-15; CellML author: Geoffrey Nunns.

Table S3. Kinetic parameters of the transport models.

Transport model	Parameter	Parameter value
**Myoplasmic compartment ⇄ Extracellular compartment**		
**Monocarboxylate Transport, MCT4**	$V_{\mathrm{max}}^{\mathrm{MCT}}$	$0.088\,\mathrm{M/min}$ [c]
	K_{LAC}	$16 \times 10^{-3}\,\mathrm{M}$[b]
	K_{H}	$10^{-8.89}$ M[a]
**CO_2 diffusion**	$\Phi_{\mathrm{diff}}^{\mathrm{CO_2}}$	25 /min [c]
**Extracellular compartment → Capillary compartment**		
**CO_2 washout**	$\Phi_{\mathrm{washout}}^{\mathrm{CO_2}}$	15 /min[c]
**HCO_3^- washout**	$\Phi_{\mathrm{washout}}^{\mathrm{HCO_3^-}}$	1.5 /min[c]

[a] value was taken from Vinnakota et al. (2010). Available from: `https://models.physiomeproject.org/exposure/aa821a8dda7e55888bd486f8cbb68791/vinnakota_rusk_palmer_shankland_kushmeric_2010_edl.cellml/view`; Accessed: 2025-01-15; CellML author: Geoffrey Nunns.
[b] reported K_m values for lactate in skeletal muscle are between 13 mM to 40 mM for MCT4 (Dimmer et al., 2000; Bonen, 2001; Halestrap and Price, 1999).
[c] heuristically adjusted.

$$\frac{d\,[\mathrm{PCr}]}{dt} = \left(-J^{\mathrm{CK}}\right)/\nu_{\mathrm{my}}^{\mathrm{w}} \tag{S36}$$

$$\frac{d\,[\mathrm{Cr}]}{dt} = \left(J^{\mathrm{CK}}\right)/\nu_{\mathrm{my}}^{\mathrm{w}} \tag{S37}$$

$$\frac{d\,[\mathrm{G1P}]}{dt} = 0\ ,\ \text{(clamped)} \tag{S38}$$

$$\frac{d\,[\mathrm{G6P}]}{dt} = \left(J^{\mathrm{PGLM}} - J^{\mathrm{PGI}}\right)/\nu_{\mathrm{my}}^{\mathrm{w}} \tag{S39}$$

$$\frac{d\,[\mathrm{F6P}]}{dt} = \left(J^{\mathrm{PGI}} - J^{\mathrm{PFK}}\right)/\nu_{\mathrm{my}}^{\mathrm{w}} \tag{S40}$$

$$\frac{d\,[\mathrm{FBP}]}{dt} = \left(J^{\mathrm{PFK}} - J^{\mathrm{FBA}}\right)/\nu_{\mathrm{my}}^{\mathrm{w}} \tag{S41}$$

$$\frac{d\,[\mathrm{GAP}]}{dt} = \left(J^{\mathrm{FBA}} - J^{\mathrm{TPI}} - J^{\mathrm{GAPDH}}\right)/\nu_{\mathrm{my}}^{\mathrm{w}} \tag{S42}$$

$$\frac{d\,[\mathrm{DHAP}]}{dt} = \left(J^{\mathrm{FBA}} + J^{\mathrm{TPI}} + J^{\mathrm{G3PDH}}\right)/\nu_{\mathrm{my}}^{\mathrm{w}} \tag{S43}$$

$$\frac{d\,[\mathrm{BPG}]}{dt} = \left(J^{\mathrm{GAPDH}} - J^{\mathrm{PGK}}\right)/\nu_{\mathrm{my}}^{\mathrm{w}} \tag{S44}$$

$$\frac{d\,[\mathrm{PG3}]}{dt} = \left(J^{\mathrm{PGK}} - J^{\mathrm{PGYM}}\right)/\nu_{\mathrm{my}}^{\mathrm{w}} \tag{S45}$$

$$\frac{d\,[\mathrm{PG2}]}{dt} = \left(J^{\mathrm{PGYM}} - J^{\mathrm{ENO}}\right)/\nu_{\mathrm{my}}^{\mathrm{w}} \tag{S46}$$

$$\frac{d\,[\mathrm{PEP}]}{dt} = \left(J^{\mathrm{ENO}} - J^{\mathrm{PYK}}\right)/\nu_{\mathrm{my}}^{\mathrm{w}} \tag{S47}$$

$$\frac{d\,[\mathrm{PYR}]}{dt} = \left(J^{\mathrm{PYK}} - J^{\mathrm{LDH}} - J^{\mathrm{OxPhos}}\right)/\nu_{\mathrm{my}}^{\mathrm{w}} \tag{S48}$$

$$\frac{d\,[\mathrm{LAC}]}{dt} = \left(J^{\mathrm{LDH}} - J_{\mathrm{my}\rightleftarrows\mathrm{ex}}^{\mathrm{MCT}} \cdot \frac{\nu_{\mathrm{ex}}^{\mathrm{r}}}{\nu_{\mathrm{my}}^{\mathrm{r}}}\right)/\nu_{\mathrm{my}}^{\mathrm{w}} \tag{S49}$$

$$\frac{d\,[\mathrm{G3P}]}{dt} = \left(-J^{\mathrm{G3PDH}}\right)/\nu_{\mathrm{my}}^{\mathrm{w}} \tag{S50}$$

$$\frac{d\,[\mathrm{NAD}]}{dt} = \left(-J^{\mathrm{GAPDH}} - J^{\mathrm{G3PDH}} + J^{\mathrm{LDH}} + J^{\mathrm{OxPhos}}\right)/\nu_{\mathrm{my}}^{\mathrm{w}} \tag{S51}$$

$$\frac{d\,[\mathrm{NADH}]}{dt} = \left(J^{\mathrm{GAPDH}} + J^{\mathrm{G3PDH}} - J^{\mathrm{LDH}} - J^{\mathrm{OxPhos}}\right)/\nu^{\mathrm{w}}_{\mathrm{my}} \tag{S52}$$

$$\frac{d\,[\mathrm{O_2}]}{dt} = 0\ ,\ \text{(clamped)} \tag{S53}$$

$$\frac{d\,[\mathrm{CO_2}]}{dt} = \left(3\cdot J^{\mathrm{OxPhos}} - J^{\mathrm{CA}} - J^{\mathrm{CO_2}}_{\mathrm{my}\rightleftarrows\mathrm{ex}}\cdot\frac{\nu^{\mathrm{r}}_{\mathrm{ex}}}{\nu^{\mathrm{r}}_{\mathrm{my}}}\right)/\nu^{\mathrm{w}}_{\mathrm{my}} \tag{S54}$$

$$\frac{d\,\left[\mathrm{HCO_3^-}\right]}{dt} = \left(J^{\mathrm{CA}}\right)/\nu^{\mathrm{w}}_{\mathrm{my}} \tag{S55}$$

Extracellular compartment

$$\frac{d\,[\mathrm{CO_2}]_{\mathrm{ex}}}{dt} = \left(-J^{\mathrm{CO_2Hyd}} + J^{\mathrm{CO_2}}_{\mathrm{my}\rightleftarrows\mathrm{ex}} - J^{\mathrm{CO_2}}_{\mathrm{ex}\rightleftarrows\mathrm{cap}}\cdot\frac{\nu^{\mathrm{r}}_{\mathrm{cap}}}{\nu^{\mathrm{r}}_{\mathrm{ex}}}\right)/\nu^{\mathrm{w}}_{\mathrm{ex}} \tag{S56}$$

$$\frac{d\,\left[\mathrm{HCO_3^-}\right]_{\mathrm{ex}}}{dt} = \left(J^{\mathrm{CO_2Hyd}} - J^{\mathrm{HCO_3^-}}_{\mathrm{ex}\rightleftarrows\mathrm{cap}}\cdot\frac{\nu^{\mathrm{r}}_{\mathrm{cap}}}{\nu^{\mathrm{r}}_{\mathrm{ex}}}\right)/\nu^{\mathrm{w}}_{\mathrm{ex}} \tag{S57}$$

The extracellular lactate concentration is clamped by default:

$$\frac{d\,[\mathrm{LAC}]_{\mathrm{ex}}}{dt} = 0\ ,\ \text{(clamped)} \tag{S58a}$$

The following equation applies for the simulations under ischaemic conditions:

$$\frac{d\,[\mathrm{LAC}]_{\mathrm{ex}}}{dt} = \left(J^{\mathrm{MCT}}_{\mathrm{my}\rightleftarrows\mathrm{ex}}\right)/\nu^{\mathrm{w}}_{\mathrm{ex}} \tag{S58b}$$

Capillary compartment

$$\frac{d\,[\mathrm{CO_2}]_{\mathrm{ex}}}{dt} = \left(-J^{\mathrm{CO_2Hyd}} + J^{\mathrm{CO_2}}_{\mathrm{my}\rightleftarrows\mathrm{ex}} - J^{\mathrm{CO_2}}_{\mathrm{ex}\rightleftarrows\mathrm{cap}}\cdot\frac{\nu^{\mathrm{r}}_{\mathrm{cap}}}{\nu^{\mathrm{r}}_{\mathrm{ex}}}\right)/\nu^{\mathrm{w}}_{\mathrm{ex}} \tag{S59}$$

$$\frac{d\,\left[\mathrm{HCO_3^-}\right]_{\mathrm{ex}}}{dt} = \left(J^{\mathrm{CO_2Hyd}} - J^{\mathrm{HCO_3^-}}_{\mathrm{ex}\rightleftarrows\mathrm{cap}}\cdot\frac{\nu^{\mathrm{r}}_{\mathrm{cap}}}{\nu^{\mathrm{r}}_{\mathrm{ex}}}\right)/\nu^{\mathrm{w}}_{\mathrm{ex}} \tag{S60}$$

S1.3 Baseline concentrations

The resting baseline concentration of each metabolite in the model is listed in Table S4. The table also contains reported concentrations in resting skeletal muscles from the literature. However, note that the reported values are a mix of biopsy data and in vivo ^{31}P-MRS data collected from different FT skeletal muscles in different species and are intended as a rough guide only.

Table S4. Basal concentrations in the model versus reported values.

Metabolite	Model (mM)[a]	Reported values (mM)[b]	Reference
**Myoplasmic compartment**			
G1P	0.07*	0.01–0.24	[1-5]
G6P	0.8	0.28–0.84	[1-6]
F6P	0.23	0.05–0.23	[1-3,5,7]
FBP	1.0×10^{-3}	40×10^{-3}–120×10^{-3}	[2-3,5]
DHAP	0.058	0.004–0.12	[3,5,8]
G3P	0.59	0.13–0.35	[1,6]
GAP	0.003	0.04	[3,7]
13BPG	0.003	0.065[c]	[7]
3PG	0.26×10^{-3}	6.6×10^{-3}–56.3×10^{-3}	[7-8]
2PG	0.08×10^{-3}	5×10^{-3}[c]	[7]
PEP	0.33×10^{-3}	19.4×10^{-3}	[7]
PYR	0.022	0.022–0.17	[1-3,5-6,8-9]
LAC	0.37	0.36–2.23	[1-4,6,8-9]
ATP	10.3	8.0–11.4	[1-6,9-12]
ADP	0.023	0.009–1.79	[1-3,5-6,9,11]
AMP	0.03×10^{-3}	0.02×10^{-3}–0.105	[1,3,5-7]
Pi	2.9	0–4.7	[5,9-12]
PCr	34.97	30.1–43.9	[1-5,6,9-12]
Cr	10.2	1.3–22.6	[1,6,8,11]
NAD/NADH	0.85/0.002 = 421	294–684	[13-15]
$CO_{2,aq}$	8.8	18.6	[16]
$CO_{2,g}$	1.67	1.63	[16]
H^+	$10^{-7.05}$	$10^{-7.02} - 10^{-7.2}$	[9-11,17]
K^+	120*	100–168	[18-19]
Mg^{2+}	1.06	0.58–1	[8-9]
**Extracellular compartment**			
LAC	1.0*	1.0–1.92	[20-21]
$CO_{2,aq}$	30	-	-
$CO_{2,g}$	1.64	-	-
H^+	$10^{-7.4}$*	$10^{-7.23}$-$10^{-7.4}$	[22]
K^+	120	-	-
Mg^{2+}	2.1	-	-

[a] M = mol/(litre cytoplasmic water).
[b] If necessary, a factor of 3.8 (Vinnakota et al., 2010) was used as the wet-to-dry weight ratio, and a factor of 0.66 (Desmedt, 1953) was used to account for the fraction of intracellular water per unit wet weight of muscle for the unit conversion into Molar.
[c] Concentration was estimated by the order of magnitude reported for other cell types (Lambeth and Kushmerick, 2002).
* clamped value.

[1] Spriet (1989), [2] Cheetham et al. (1986), [3] Harris et al. (1974), [4] Blomastrand et al. (1985) , [5] Green et al. (1995), [6] Sahlin et al. (1987), [7] Lambeth and Kushmerick (2002), [8] Connett (1985), [9] Cieslar and Dobson (2000), [10] Kushmerick and Meyer (1985), [11] Jeneson et al. (1995), [12] Kushmerick et al. (1992), [13] White and Schenk (2012), [14] Graham et al. (1986), [15] Glancy et al. (2021), [16] Veech et al. (1979), [17] Foley et al. (1991),[18] Sjogaard et al. (1985), [19] Sejersted and Sjøgaard (2000), [20] de Boer et al. (1991), [21] Brooks (2018), [22] Hosonuma and Yoshimura (2023)

S2 MPSA

A local multi-parameter sensitivity analysis (MPSA) was conducted with the algorithm summarized in the following (for further details see Hornberger and Spear (1981); Chang and Delleur (1992); Choi et al. (1998); Zi et al. (2005)):

(1) A total of 91 parameters were tested. 85 kinetic parameters, 20 of which are V^k_{max} values, plus the basal pH level and 5 metabolite pools (total phosphate pool, TPP; total creatine pool, TCr; total adenine nucleotide pool, TAN; total NAD and NADH redox pool, redox; total generic buffer size, BX). The apparent equilibrium constants $K^k_{\text{app}}(\text{pH})$ of the enzyme reactions are computed as a function of pH and were not included.

(2) The perturbation range for the tested parameters was set to $\pm 1\,\%$.

(3) A Latin hypercube sampling method was used to generate 3000 parameter sets drawn from the given parameter range. The distributions of the parameter values in an intact physiological system are unknown; we thus assumed a uniform parameter distribution. The sampling method was implemented using the MATLAB function 'lhsdesign'. The random number generator was initialized using the default algorithm and seed.

(4) The model was run with each generated parameter vector $\boldsymbol{p}$ simulating three exercise intensities following a protocol of a 5 min resting phase with basal ATP hydrolysis rate, a 5 min exercise phase with an ATPase rate above basal level (low intensity: ATPase = 10 mM/min; medium intensity: ATPase = 20 mM/min; high intensity: ATPase = 40 mM/min) and a subsequent 5 min recovery phase at basal ATPase rate. An initialization phase was implemented so that the model could reach a steady-state baseline before the simulation protocol started. The pH level was clamped during the initialization phase. To evaluate the effect of a parameter perturbation on the simulation output with respect to 10 selected state variables m ([Pi], [PCr], pH, [ATP], [ADP], [AMP], [FBP], [G3P], [PYR] and [LAC]), a loss function was defined as the sum of squared errors between the simulation with the reference parametrization $x^j_m(t_i, \boldsymbol{p}_{\text{ref}})$ and the simulations with the randomly generated parameter sets $x^j_m(t_i, \boldsymbol{p})$:

$$L^j_m(\boldsymbol{p}) = \sum_{i=1}^{N_i} \Big(x^j_m(t_i, \boldsymbol{p}) - x^j_m(t_i, \boldsymbol{p}_{\text{ref}}) \Big)^2 \, . \tag{S61}$$

Therein, N_i denotes the number of time samples t_i. The loss function value for each parameter set $\boldsymbol{p}$ with respect to the mth selected state variable was calculated for the metabolite baseline ($j = 1$: basal ATPase rate, i.e. 0.48 mM/min) and the three dynamic responses, combining the exercise phase at a different ATPase rate, respectively, and the subsequent recovery phase ($j = 2, 3, 4$). For the evaluation of the dynamic response, the baseline of the reference simulation and the perturbed simulation results were shifted to zero.

(5) The parameter sets $\boldsymbol{p}$ were classified to be either acceptable or unacceptable through thresholding. The mean loss function value (considering all 3000 simulations with different parameter sets) for each metabolic state variable was used as the corresponding threshold. Note that there are separate thresholds for the evaluation of the resting state sensitivity (mean loss function value over all simulation runs at rest) and for the evaluation of the dynamic response of the model at each level of muscle activation (mean loss function value over all simulation runs during the active and recovery phase combined). A parameter set $\boldsymbol{p}$ is acceptable in reference to one of the metabolic state variables (at working rate j) if the loss function value is below the associated threshold value and unacceptable if the value is above.

(6) For each tested parameter, the cumulative distribution functions of the parameter values from the accepted and unaccepted parameter sets were evaluated using a Kolmogorov-Smirnov (K-S) statistic

given as:

$$D^j_{m,n} = \sup_{q_n} \left| F^j_{a,m}(p_n) - F^j_{u,m}(p_n) \right| \tag{S62}$$

where $F^j_{a,m}$ and $F^j_{u,m}$ are the cumulative distribution functions of the accepted and unaccepted parameter values, respectively, of a tested parameter with respect to the mth metabolic state variable at work rate j. The K-S statistic determines the largest absolute vertical difference between the two distribution functions. Consequently, if the K-S value ($D_{m,n}$) is large, the mth state variable is classified as sensitive to changes of the nth tested parameter.

References

Blei, M., Conley, K., and Kushmerick, M. (1993). Separate measures of atp utilization and recovery in human skeletal muscle. *The Journal of physiology*, 465(1):203–222.

Blomastrand, E., Larsson, L., and Edstrom, L. (1985). Contractile properties, fatiguability and glycolytic metabolism in fast-and slow-twitch rat skeletal muscles of various temperatures. *Acta physiologica scandinavica*, 125(2):235–243.

Bonen, A. (2001). The expression of lactate transporters (mct1 and mct4) in heart and muscle. *European journal of applied physiology*, 86:6–11.

Brooks, G. A. (2018). The science and translation of lactate shuttle theory. *Cell metabolism*, 27(4):757–785.

Chance, B. and Connelly, C. (1957). A method for the estimation of the increase in concentration of adenosine diphosphate in muscle sarcosomes following a contraction. *Nature*, 179(4572):1235–1237.

Chang, F.-J. and Delleur, J. (1992). Systematic parameter estimation of watershed acidification model. *Hydrological Processes*, 6(1):29–44.

Cheetham, M. E., Boobis, L., Brooks, S., and Williams, C. (1986). Human muscle metabolism during sprint running. *Journal of applied physiology*, 61(1):54–60.

Choi, J., Hulseapple, S., Conklin, M., and Harvey, J. (1998). Modeling co2 degassing and ph in a stream–aquifer system. *Journal of Hydrology*, 209(1-4):297–310.

Cieslar, J. H. and Dobson, G. P. (2000). Free [adp] and aerobic muscle work follow at least second order kinetics in rat gastrocnemius in vivo. *Journal of Biological Chemistry*, 275(9):6129–6134.

Connett, R. J. (1985). In vivo glycolytic equilibria in dog gracilis muscle. *Journal of Biological Chemistry*, 260(6):3314–3320.

Connett, R. J. (1989). In vivo control of phosphofructokinase: system models suggest new experimental protocols. *American Journal of Physiology-Regulatory, Integrative and Comparative Physiology*, 257(4):R878–R888.

Crow, M. T. and Kushmerick, M. J. (1982). Chemical energetics of slow-and fast-twitch muscles of the mouse. *The Journal of general physiology*, 79(1):147–166.

de Boer, J., Postema, F., Plijter-Groendijk, H., and Korf, J. (1991). Continuous monitoring of extracellular lactate concentration by microdialysis lactography for the study of rat muscle metabolism in vivo. *Pflügers Archiv*, 419:1–6.

Desmedt, J. E. (1953). Electrical activity and intracellular sodium concentration in frog muscle. *The Journal of Physiology*, 121(1):191.

Dimmer, K.-S., Friedrich, B., Lang, F., Deitmer, J. W., and Bröer, S. (2000). The low-affinity monocarboxylate transporter mct4 is adapted to the export of lactate in highly glycolytic cells. *Biochemical Journal*, 350(1):219–227.

Foley, J. M., Harkema, S. J., and Meyer, R. A. (1991). Decreased atp cost of isometric contractions in atp-depleted rat fast-twitch muscle. *American Journal of Physiology-Cell Physiology*, 261(5):C872–C881.

Glancy, B., Kane, D. A., Kavazis, A. N., Goodwin, M. L., Willis, W. T., and Gladden, L. B. (2021). Mitochondrial lactate metabolism: history and implications for exercise and disease. *The Journal of physiology*, 599(3):863–888.

Graham, T., Barclay, J., and Wilson, B. (1986). Skeletal muscle lactate release and glycolytic intermediates during hypercapnia. *Journal of Applied Physiology*, 60(2):568–575.

Green, H., Jones, S., Ball-Burnett, M., Farrance, B., and Ranney, D. (1995). Adaptations in muscle metabolism to prolonged voluntary exercise and training. *Journal of Applied Physiology*, 78(1):138–145.

Halestrap, A. P. (1975). The mitochondrial pyruvate carrier. kinetics and specificity for substrates and inhibitors. *Biochemical Journal*, 148(1):85–96.

Halestrap, A. P. and Price, N. T. (1999). The proton-linked monocarboxylate transporter (mct) family: structure, function and regulation. *Biochemical Journal*, 343(2):281–299.

Harris, R., Hultman, E., and Nordesjö, L.-O. (1974). Glycogen, glycolytic intermediates and high-energy phosphates determined in biopsy samples of musculus quadriceps femoris of man at rest. methods and variance of values. *Scandinavian journal of clinical and laboratory investigation*, 33(2):109–120.

Hornberger, G. M. and Spear, R. C. (1981). Approach to the preliminary analysis of environmental systems. *J. Environ. Mgmt.*, 12(1):7–18.

Hosonuma, M. and Yoshimura, K. (2023). Association between ph regulation of the tumor microenvironment and immunological state. *Frontiers in Oncology*, 13:1175563.

Jeneson, J., Westerhoff, H., Brown, T., Van Echteld, C., and Berger, R. (1995). Quasi-linear relationship between gibbs free energy of atp hydrolysis and power output in human forearm muscle. *American Journal of Physiology-Cell Physiology*, 268(6):C1474–C1484.

Kushmerick, M. and Meyer, R. (1985). Chemical changes in rat leg muscle by phosphorus nuclear magnetic resonance. *American Journal of Physiology-Cell Physiology*, 248(5):C542–C549.

Kushmerick, M. J., Moerland, T. S., and Wiseman, R. W. (1992). Mammalian skeletal muscle fibers distinguished by contents of phosphocreatine, atp, and pi. *Proceedings of the National Academy of Sciences*, 89(16):7521–7525.

Lambeth, M. J. and Kushmerick, M. J. (2002). A computational model for glycogenolysis in skeletal muscle. *Annals of biomedical engineering*, 30(6):808–827.

LaNoue, K. F. and Schoolwerth, A. C. (1979). Metabolite transport in mitochondria. *Annual review of biochemistry*, 48(1):871–922.

Sahlin, K., Katz, A., and Henriksson, J. (1987). Redox state and lactate accumulation in human skeletal muscle during dynamic exercise. *Biochemical Journal*, 245(2):551–556.

Schantz, P., Sjöberg, B., and Svedenhag, J. (1986). Malate-aspartate and alpha-glycerophosphate shuttle enzyme levels in human skeletal muscle: methodological considerations and effect of endurance training. *Acta physiologica scandinavica*, 128(3):397–407.

Schmitz, J. P., Groenendaal, W., Wessels, B., Wiseman, R. W., Hilbers, P. A., Nicolay, K., Prompers, J. J., Jeneson, J. A., and van Riel, N. (2013). Combined in vivo and in silico investigations of activation of glycolysis in contracting skeletal muscle. *American Journal of Physiology-Cell Physiology*, 304(2):C180–C193.

Sejersted, O. M. and Sjøgaard, G. (2000). Dynamics and consequences of potassium shifts in skeletal muscle and heart during exercise. *Physiological reviews*, 80(4):1411–1481.

Sjogaard, G., Adams, R. P., and Saltin, B. (1985). Water and ion shifts in skeletal muscle of humans with intense dynamic knee extension. *American Journal of Physiology-Regulatory, Integrative and Comparative Physiology*, 248(2):R190–R196.

Spriet, L. L. (1989). Atp utilization and provision in fast-twitch skeletal muscle during tetanic contractions. *American Journal of Physiology-Endocrinology and Metabolism*, 257(4):E595–E605.

Vanlier, J., Wu, F., Qi, F., Vinnakota, K. C., Han, Y., Dash, R. K., Yang, F., and Beard, D. A. (2009). Bisen: Biochemical simulation environment. *Bioinformatics*, 25(6):836–837.

Veech, R. L., Lawson, J., Cornell, N., and Krebs, H. A. (1979). Cytosolic phosphorylation potential. *Journal of Biological Chemistry*, 254(14):6538–6547.

Vinnakota, K., Kemp, M. L., and Kushmerick, M. J. (2006). Dynamics of muscle glycogenolysis modeled with ph time course computation and ph-dependent reaction equilibria and enzyme kinetics. *Biophysical journal*, 91(4):1264–1287.

Vinnakota, K. C., Rusk, J., Palmer, L., Shankland, E., and Kushmerick, M. J. (2010). Common phenotype of resting mouse extensor digitorum longus and soleus muscles: equal atpase and glycolytic flux during transient anoxia. *The Journal of physiology*, 588(11):1961–1983.

Vinnakota, K. C., Wu, F., Kushmerick, M. J., and Beard, D. A. (2009). Multiple ion binding equilibria, reaction kinetics, and thermodynamics in dynamic models of biochemical pathways. *Methods in enzymology*, 454:29–68.

Waser, M. R., Garfinkel, L., Kohn, M. C., and Garfinkel, D. (1983). Computer modeling of muscle phosphofructokinase kinetics. *Journal of Theoretical Biology*, 103(2):295–312.

Watt, I. N., Montgomery, M. G., Runswick, M. J., Leslie, A. G., and Walker, J. E. (2010). Bioenergetic cost of making an adenosine triphosphate molecule in animal mitochondria. *Proceedings of the National Academy of Sciences*, 107(39):16823–16827.

White, A. T. and Schenk, S. (2012). Nad+/nadh and skeletal muscle mitochondrial adaptations to exercise. *American Journal of Physiology-Endocrinology and Metabolism*, 303(3):E308–E321.

Zi, Z., Cho, K.-H., Sung, M.-H., Xia, X., Zheng, J., and Sun, Z. (2005). In silico identification of the key components and steps in ifn-γ induced jak-stat signaling pathway. *FEBS letters*, 579(5):1101–1108.
